# Long-term labelling and tracing of endodermal cells using a perpetual cycling Gal4-UAS system

**DOI:** 10.1242/dev.204289

**Published:** 2025-03-21

**Authors:** Yanfeng Li, You Li, Bangzhuo Huang, Ruhao Zhang, Jianbo He, Lingfei Luo, Yun Yang

**Affiliations:** ^1^ Institute of Developmental Biology and Regenerative Medicine, Southwest University, Beibei 400715, Chongqing, China; ^2^ School of Life Sciences, Fudan University, Shanghai 200438, China

**Keywords:** Gal4-UAS system, Endoderm, Cell labelling, Lineage tracing, Zebrafish

## Abstract

Cell labelling and lineage tracing are indispensable tools in developmental biology, offering powerful means with which to visualise and understand the complex dynamics of cell populations during embryogenesis. Traditional cell labelling relies heavily on signal stability, promoter strength and stage specificity, limiting its application in long-term tracing. In this report, we optimise and reconfigure a perpetual cycling Gal4-UAS system employing a previously unreported Gal4 fusion protein and the autoregulatory Gal4 expression loop. As validated through heat-shock induction, this configuration ensures sustained transcription of reporter genes in target cells and their descendant cells while minimising cytotoxicity, thereby achieving long-term labelling and tracing. Further exploiting this system, we generate zebrafish transgenic lines with continuous fluorescent labelling specific to the endoderm, and demonstrate its effectiveness in long-term tracing by showing the progression of endoderm development from embryo to adult, providing visualisation of endodermal cells and their derived tissues. This continuous labelling and tracing strategy can span the entire process of endodermal differentiation, from progenitor cells to mature functional cells, and is applicable to studying endoderm patterning and organogenesis.

## Teaser

**Summary:** A continuous fluorescent labelling strategy for long-term cell tracing, enabling effective visualisation of endodermal cells and their development from embryos to adults.

## INTRODUCTION

Cell lineage tracing stands as a foundational technique in the study of embryonic development, capable of providing crucial insights into cell fate determination, lineage differentiation, migration, morphogenesis and the intricate processes of tissue formation (Kretzschmar and Watt, 2012; Kester and van Oudenaarden, 2018; Zhang et al., 2020). Traditional cell labelling, such as the use of vital tracer dyes (Filby et al., 2015) and photoconvertible fluorescent proteins (Adam et al., 2014), often faces issues with signal attenuation, making long-term tracing of labelled cells difficult. The Cre-loxP system is a widely used tool at present for lineage tracing, providing persistent labelling of the targeted cells through irreversible genetic recombination (Zong et al., 2005; Mosimann et al., 2011; He et al., 2017). However, its efficiency relies on the strength of specific promoter driving Cre (Ma et al., 2008), the activity of constitutive promoter controlling reporter cassettes (Lalonde et al., 2022) and the distance between loxP recombination sites (Vooijs et al., 2001). Therefore, expanding stable and long-term cell labelling strategies is of significant importance for deep comprehension of embryonic development and continuous mapping of cell lineages.

The endoderm is one of the earliest progenitor cell types to form during embryonic development and, in principle, retains the ability to generate nearly all cell lineages of respiratory and digestive organs. SRY-box transcription factor 17 (*Sox17*) is a representative gene for defining the endoderm lineage, critically contributing to the formation and maintenance of endoderm in fish, mice and other species (Ober et al., 2003; Viotti et al., 2014; Mukherjee et al., 2020). In zebrafish embryos, the injection of a photoactivatable fluorescein dextran conjugate into the *Tg(sox17:GFP)* transgenic line (Chung and Stainier, 2008 and Chung et al., 2008) or the direct generation of the *Tg(sox17:hKikGR1)* transgenic line that expressed a photoconvertible Kikume fluorescent protein (Yang et al., 2021) have been used to label and trace endodermal cells to identify progenitor regions of the digestive organ in the endoderm following gastrulation. Notably, these endoderm lineage studies focus on a relatively short stage of development, due to the temporal constraints of *sox17* transcription (Alexander and Stainier, 1999), and are unable to mark subsequent differentiated cells constantly. Tracing the endodermal lineage in the long term remains an issue that needs to be addressed.

The Gal4-UAS system, initially developed in *Drosophila*, is a bipartite expression system consisting of the yeast transcription activator Gal4 and its upstream activating sequence (UAS). When Gal4 is expressed, it binds to UAS and activates the transcription of downstream genes (Brand and Perrimon, 1993; Phelps and Brand, 1998). This system has emerged as a powerful tool for controlling gene expression in model organisms, such as zebrafish (Goll et al., 2009; Kawakami et al., 2016; Zhang et al., 2019). The Gal4-VP16, a fusion of the Gal4 DNA-binding domain and the herpes simplex virion protein 16 (VP16) activation domain, has been shown to be a potent transcriptional enhancer that can increase the expression of UAS-driven downstream genes and amplify their output signals (Köster and Fraser, 2001; Asakawa et al., 2008), especially when a weak promoter is used (Iyer et al., 2001; Xiong et al., 2013). A pioneering study developed an effective strategy to achieve sustained expression of the Gal4 by an autoregulatory Gal4-UAS feedback loop (Distel et al., 2009). For these reasons, combined with fluorescent reporters, the Gal4-UAS system is well suited for tracing some progenitors whose marker gene expression is low and transient. In this Report, we have optimised and reconfigured a perpetual cycling Gal4-UAS system, utilising it to generate zebrafish transgenic lines with endoderm-specific and continuous fluorescent labelling, thereby enabling long-term tracing of endodermal cells and visualisation of their developmental process. This technique provides a valuable tool for future in-depth studies of endodermal differentiation and tissue formation.

## RESULTS AND DISCUSSION

### Achieving long-term cell labelling using a perpetual cycling Gal4-UAS system

**Fig. 1.**
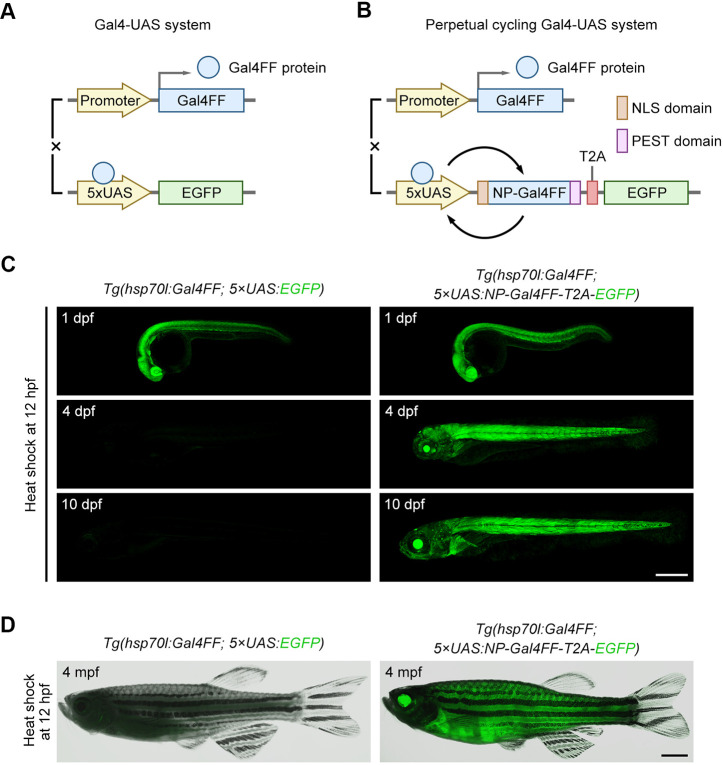
**Comparison between the traditional Gal4-UAS system and a perpetual cycling Gal4-UAS system.** (A,B) Schematic of the traditional Gal4-UAS system (A) versus a perpetual cycling Gal4-UAS system (B). Gal4FF (blue boxes) indicates the engineered yeast Gal4 transcription activator. NP-Gal4FF is composed of the Gal4FF fused with the nuclear localisation signal (NSL) domain (brown boxes) at the 5′ end and the Pro/Glu/Ser/Thr-rich (PEST) domain (purple boxes) at the 3′ end. (C,D) Comparison of fluorescence duration in two reporter systems through heat-shock induction of Gal4FF. After heat-shock at 12 h post-fertilisation (hpf), the EGFP signal in transgenic line *Tg(hsp70l:Gal4FF; 5×UAS:EGFP)* was lost by 4 days post-fertilisation (dpf), whereas the EGFP signal in the transgenic line *Tg(hsp70l:Gal4FF; 5×UAS:NP-Gal4FF-T2A-EGFP)* remained strong (C) and sustained until 4 months post-fertilisation (mpf) or adulthood (D). Scale bars: 500 µm in C; 5 mm in D.

Our perpetual cycling Gal4-UAS system is based upon the traditional Gal4-UAS system (Fig. 1A) with a series of optimisation improvements. In detail, Gal4FF is an attenuated version of the Gal4-VP16 (Asakawa and Kawakami, 2008), its protein coding sequence is linked with enhanced GFP (EGFP) via a *Thosea asigna* virus self-cleaving 2A peptide (T2A), placed the downstream of five tandem repeats of UAS (5×UAS). Once Gal4FF is driven to expression by a specific promoter, it binds to UAS and activates the transcription of Gal4FF-T2A-EGFP. The newly produced Gal4FF binds to UAS again, thus continuously cycling transcriptional activation of Gal4FF and EGFP (Fig. 1B). Considering the mutual exclusivity between the DNA-binding and nuclear-targeting activities of Gal4 (Chan et al., 1998; Chan and Jans, 2001), we incorporated a nuclear localisation signal (NLS) from simian virus 40 (SV40) large T-antigen to the 5′ end of Gal4FF protein-coding sequence, to improve its nuclear import efficiency. Meanwhile, a modified peptide sequence rich in proline, glutamic acid, serine and threonine (PEST) was added to the 3′ end (Li et al., 1998; Ravindran et al., 2022) to accelerate the degradation of Gal4FF, reducing its toxic accumulation during continuous transcriptional activation. This new NLS-Gal4FF-PEST fusion (Table S1), abbreviated as NP-Gal4FF, can increase the transcriptional potency of Gal4FF while minimising cytotoxicity.

To evaluate the above-described optimised scheme, we transiently transfected 293T cells with a tamoxifen-responsive *Gal4FF-ER^T2^* construct (Gerety et al., 2013) under the *human cytomegalovirus* (*CMV*) promoter to compare the expression levels of *5×UAS:EGFP*, *5×UAS:EGFP-PEST* and *5×UAS:NP-Gal4FF-T2A-EGFP* reporters after tamoxifen exposure (Fig. S1A). The results showed that EGFP fused with the PEST domain degraded faster than unmodified EGFP, whereas NP-Gal4FF-T2A-EGFP markedly prolonged the duration of EGFP expression through Gal4-UAS cycling transcription (Fig. S1B,C). As part of the toxicity assay, different *Gal4FF* constructs carried by the *pTol2-CMV* backbone were separately injected into one-cell stage zebrafish embryos (Fig. S2A). By 48 h post fertilisation (hpf), through comparison at the same injection dosage, the ratios of dead and deformed embryos with the *Gal4FF* constructs containing the *PEST* sequence were lower than those without it (Fig. S2B,C). Furthermore, to minimise background toxicity, we generated a heat shock-inducible Gal4FF line *Tg(hsp70l:NP-Gal4FF)^cq210^* and crossed it with different reporter lines to examine the phenotype of their offspring after heat shock (Fig. S3A). The results showed that, under the Gal4-5×UAS cycling activation, NLS-Gal4FF-PEST caused less toxic damage to embryos than NLS-Gal4FF (Fig. S3B,C). Notably, under the Gal4-10×UAS cycling activation, the ratios of dead and deformed embryos were increased significantly.

Pursuing further assessment of the efficiency and persistence of cell labelling, a basic Hsp70l-Gal4FF transgenic line was crossed with the fluorescent reporter line *Tg(5×UAS:NP-Gal4FF-T2A-EGFP)^cq187^*, which was selected through the above-optimised comparison. Their offspring were heat-shocked at 12 hpf to monitor EGFP expression during subsequent embryonic development. Within traditional Gal4-UAS systems, EGFP signal in transgenic line *Tg(hsp70l:Gal4FF; 5×UAS:EGFP)* had been depleted by 4 days post-fertilisation (dpf). By contrast, EGFP expression in the perpetual cycling Gal4-UAS system was maintained robustly for an extended period (Fig. 1C), even being able to continue into adulthood (Fig. 1D). These data validate the feasibility of this optimised scheme for the sustained expression of Gal4FF and EGFP, indicating that the perpetual cycling Gal4-UAS system can effectively accomplish stable and long-term cell labelling.

### Establishment of a perpetual cycling Gal4-UAS system specific to endoderm

**Fig. 2.**
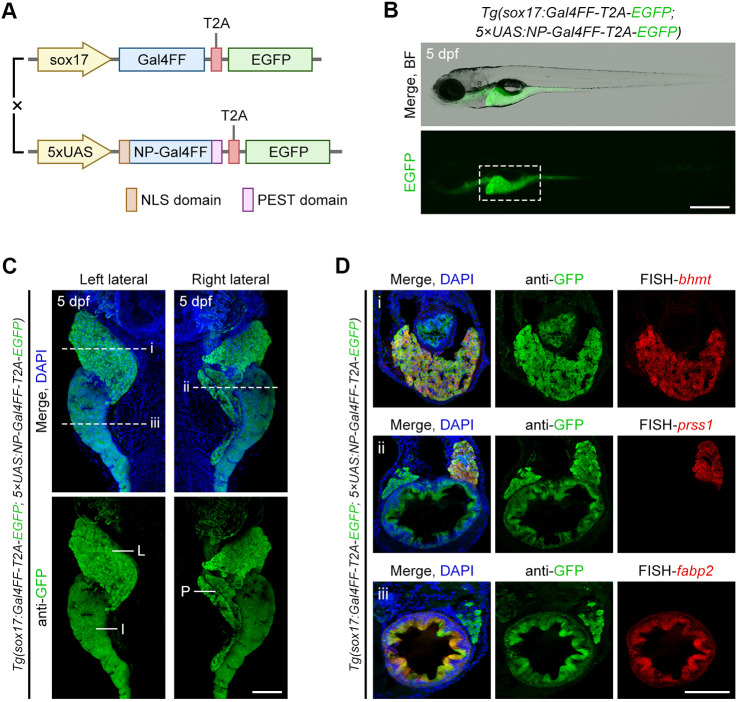
**Generation of endoderm-specific fluorescent reporter lines using a perpetual cycling Gal4-UAS system.** (A) Schematic of a perpetual cycling Gal4-UAS system for fluorescent labelling specific to endoderm. Gal4FF-T2A-EGFP expression was driven by the endoderm-specific promoter *sox17*. (B-D) Under the *Tg(sox17:Gal4FF-T2A-EGFP; 5×UAS:NP-Gal4FF-T2A-EGFP)* transgenic background, fluorescent signal was observed in endoderm-derived tissues of zebrafish larvae at 5 dpf (B). The region outlined in B corresponds to the magnified lateral views in C, showing EGFP expression in the major accessory organs of digestion. L, liver; P, pancreas; I, intestine. Dashed lines i, ii and iii in C indicate the transverse planes corresponding to the tissue sections in i, ii and iii in D, showing EGFP expression in *bhmt*+ hepatocytes (i), *prss1*+ pancreatic acinar cells (ii) and *fabp2*+ intestinal enterocytes (iii). Scale bars: 500 µm in B; 100 µm in C,D.

For applying the perpetual cycling Gal4-UAS system to endoderm lineage studies, we generated a transgenic line *Tg(sox17:Gal4FF-T2A-EGFP)^cq186^*, in which Gal4FF-T2A-EGFP expression was driven by the endoderm-specific promoter *sox17*, and crossed it with the fluorescent reporter line *Tg(5×UAS:NP-Gal4FF-T2A-EGFP)* to enable continuous fluorescent labelling of the endoderm and its descendant cells (Fig. 2A). In the process of establishing double transgenic lines, we conducted extensive and multi-round screening to identify lines with relatively stable and consistent expression of reporter genes (Fig. S4A), as numerous different founders of the *Tg(5×UAS:NP-Gal4FF-T2A-EGFP)* produce offspring with suboptimal and variable expression patterns (Fig. S4B,C). Such variability is likely caused by the position effects from random integration into the genome (Clark et al., 1994), a phenomenon particularly pronounced in our perpetual cycling Gal4-UAS system.

Within a pre-screened and stabilised double transgenic genetic background, all endoderm-derived tissues along the anterior-posterior axis, from the oral cavity to the cloaca, could be visualised (Fig. 2B), including the major accessory organs such as the liver, pancreas and intestine, as shown in the magnified lateral views of larvae at 5 dpf (Fig. 2C). Tissue sections further displayed *betaine-homocysteine methyltransferase* (*bhmt*)-expressing hepatocytes (Gao et al., 2019), *serine protease 1* (*prss1*, also known as *trypsin*)-expressing pancreatic acinar cells (Raby et al., 2021) and *fatty acid binding protein 2* (*fabp2*)-expressing intestinal enterocytes (Yang et al., 2022), all featuring noticeable EGFP signals (Fig. 2D). These data suggest that our perpetual cycling Gal4-UAS system is capable of providing effective and reliable fluorescent labelling for endoderm lineage tracing, contributing to monitoring the developmental dynamics of endoderm-derived tissues and profiling their lineage differentiation.

### Tracing endoderm development via a perpetual cycling Gal4-UAS system

**Fig. 3.**
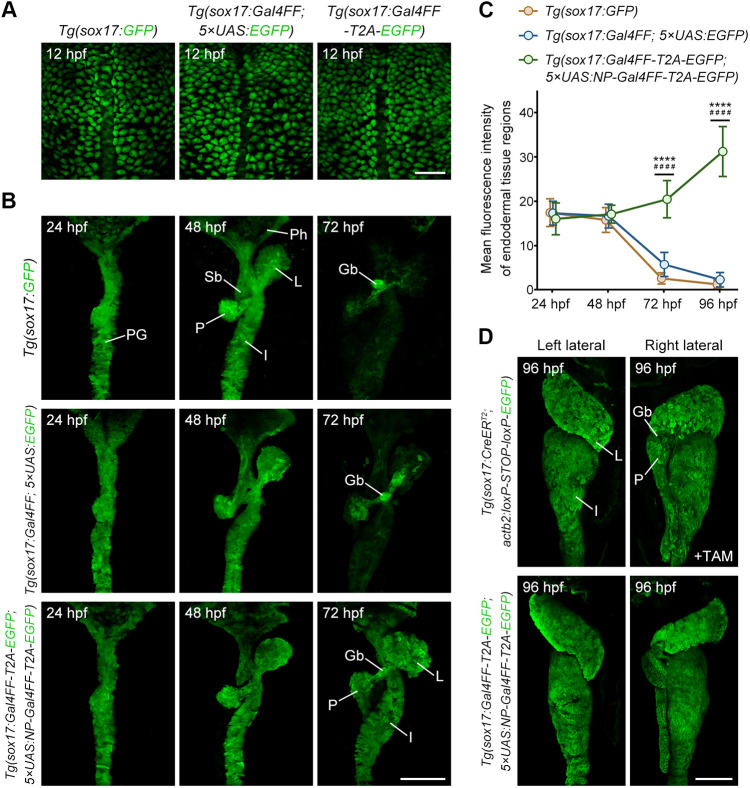
**Application of a perpetual cycling Gal4-UAS system to tracing endoderm development in zebrafish.** (A,B) Fluorescent labelling specific to endoderm using three types of transgenic lines at different stages of development. In *Tg(sox17:Gal4FF-T2A-EGFP; 5×UAS:NP-Gal4FF-T2A-EGFP)* lines, EGFP expression was maintained robustly in endodermal tissues from 12 hpf to 72 hpf, whereas in *Tg(sox17:GFP)* and *Tg(sox17:Gal4FF; 5×UAS:EGFP)* lines, fluorescent signal were retained only in the gallbladder and some surrounding areas by 72 hpf. (C) Mean fluorescence intensity of endodermal tissue regions in three types of transgenic lines at different stages of development (per group *n*=35). Data are mean±s.d. ****P<0.0001, *Tg(sox17:Gal4FF-T2A-EGFP; 5×UAS:NP-Gal4FF-T2A-EGFP)* versus *Tg(sox17:Gal4FF; 5×UAS:EGFP)*; ^####^*P*<0.0001, *Tg(sox17:Gal4FF-T2A-EGFP; 5×UAS:NP-Gal4FF-T2A-EGFP)* versus *Tg(sox17:GFP)* (one-way ANOVA with Tukey's multiple comparisons test). (D) Expression pattern of EGFP in *Tg(sox17:CreER^T2^; actb2:loxP-stop-loxP-EGFP)* and *Tg(sox17:Gal4FF-T2A-EGFP; 5×UAS:NP-Gal4FF-T2A-EGFP)* lines at 96 hpf were comparable. Tamoxifen treatment (+TAM) of embryos was initiated at 2 hpf and continued for 24 h, with analysis at 96 hpf. PG, primitive gut; Ph, pharynx; L, liver; Sb, swimbladder; P, pancreas; I, intestine; Gb, gallbladder. Scale bars: 100 µm.

Since *sox17* expression begins in gastrulation and gradually disappears in somitogenesis (Alexander and Stainier, 1999), the *Tg(sox17:GFP)* transgenic line provides only short-term labelling of endodermal tissues within a particular temporal window. To further validate the long-term continuity and reliability of endoderm labelling in our perpetual cycling Gal4-UAS system, we compared it with the single transgenic line *Tg(sox17:GFP)* and the double transgenic line *Tg(sox17:Gal4FF; 5×UAS:EGFP)*, which was established through the traditional Gal4-UAS system. The time-course analysis of embryonic development showed that, at 12 hpf, the post-gastrulation stage, *sox17*-positive endodermal cells in three comparative groups were dispersed across the embryo in a relatively sparse pattern (Fig. 3A). At 24 hpf, these cells formed the primitive gut tube, without detectable differences between groups. During the subsequent 48 hpf, multiple organ primordia, including the pharyngeal arches, liver, swim bladder, pancreas and intestine, had developed and extended from the primitive gut tube. Until 72 hpf, the fluorescence signal in gut endoderm of *Tg(sox17:GFP)* and *Tg(sox17:Gal4FF; 5×UAS:EGFP)* transgenic lines drastically decayed, except in the gallbladder and its surrounding tissues, because *sox17* transcription was re-initiated there (Shin et al., 2012). In comparison, endodermal cells labelled using the perpetual cycling Gal4-UAS system maintained robust EGFP expression (Fig. 3B), corroborated by measuring the mean fluorescence intensity of endodermal tissue regions (Fig. 3C). These results demonstrate the effectiveness of a perpetual cycling Gal4-UAS system for labelling and tracing endoderm lineage.

In addition, we conducted comparative analyses between the conventional Cre-loxP system and the perpetual cycling Gal4-UAS system. The transgenic lines *Tg(actb2:loxP-STOP-loxP-EGFP)^cq214^* (*actb2*, also known as *β-actin2*) and *Tg(5×UAS:NP-Gal4FF-T2A-EGFP)* were individually crossed with the same heat-shock line *Tg(hsp70l:Cre-P2A-Gal4FF)^cq211^* for simultaneous activation of Cre and Gal4FF after heat shock. At 24 hpf, through analysis under different heat-shock durations (Fig. S5A), the perpetual cycling Gal4-UAS system had the advantage of rapidly amplifying fluorescent signals over a short time, whereas the Cre-loxP system displayed relatively latency (Fig. S5B,C). Within an endoderm-specific *Tg(sox17:CreER^T2^)^cq213^* genetic background (Fabian et al., 2020), the Cre-loxP system following tamoxifen exposure irreversibly labelled endoderm-derived organs, including the liver, pancreas and intestine, when observed at 4 dpf, while the perpetual cycling Gal4-UAS system also demonstrated comparable capability (Fig. 3D). Notably, no obvious fluorescent signal was detected in the endoderm cells at 12 hpf, after tamoxifen treatment of *Tg(sox17:CreER^T2^; actb2:loxP-STOP-loxP-EGFP)* lines (Fig. S5D), indicating that this system relies on the activity of the *actb2* promoter as well as *sox17*. Due to the maternal contribution of *actb2* during early embryogenesis (Pinto et al., 2020; Lalonde et al., 2022), its transcription initiation in endodermal cells may occur relatively late. In comparison, this issue was not present in *Tg(sox17:Gal4FF; 5×UAS:NP-Gal4FF-T2A-EGFP)* lines (Fig. S5D), suggesting the perpetual cycling Gal4-UAS system can timely label endodermal cells without being restricted by constitutive promoters, facilitating the visualisation and tracing of these cells at earlier embryonic stages.

### Fluorescent labelling of endoderm-derived organs continues into adulthood

**Fig. 4.**
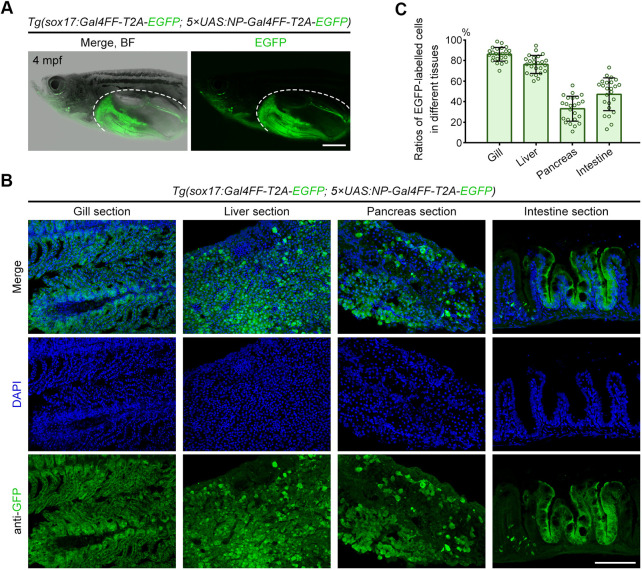
**Fluorescence detection in endoderm-derived organs of adult zebrafish harbouring a perpetual cycling Gal4-UAS system.** (A) Under the *Tg(sox17:Gal4FF-T2A-EGFP; 5×UAS:NP-Gal4FF-T2A-EGFP)* transgenic background, the fluorescent signal was observed in the respiratory and digestive organs of adult zebrafish at 4 mpf. Dashed outlines indicate the visceral region after laparotomy. Scale bar: 5 mm. (B) Tissue sections displayed EGFP expression in the gill, liver, pancreas and intestine. Scale bar: 100 µm. (C) The ratios of EGFP-labelled cells in these tissues (per group *n*=24). Data are mean±s.d.

Upon completing the analysis of the *Tg(sox17:Gal4FF-T2A-EGFP; 5×UAS:NP-Gal4FF-T2A-EGFP)* transgenic larvae, we next examined whether the perpetual cycling Gal4-UAS system can continuously label endodermal tissues and organs into adulthood. After laparotomy of adult fish at 4 months post-fertilisation (mpf), EGFP signals remained observable in the aero-digestive tract and its accessory organs derived from endoderm (Fig. 4A). Tissue sections further displayed that, although the fluorescent labelling of these organs was not as uniform as during the larval stage, a notable fraction of cells within them were continuously labelled by EGFP (Fig. 4B). Statistically, the gill and liver exhibited higher ratios of labelled cells, while the pancreas and intestine had relatively lower ratios (Fig. 4C). One possible reason for these discrepancies is, as recorded in previous studies, the faster rates of cellular turnover in the pancreas and intestine under natural conditions (Williams et al., 2015; Zhao et al., 2023), particularly in comparison to the liver (Duncan et al., 2009). This constant cell renewal can lead to increased susceptibility to DNA methylation, silencing UAS-driven transgene expression over time (Halpern et al., 2008; Goll et al., 2009). Despite the uneven labelling efficiency and evident tissue-specific differences in adult fish, these data still confirm that the perpetual cycling Gal4-UAS system has the ability to label endodermal tissues and organs into adulthood.

In summary, we showcase here a continuous fluorescent labelling strategy that utilises the optimised Gal4-UAS system to allow the perpetual cycling transcription of reporter genes, which is available for long-term cell lineage tracing. Within this strategy, the improved NP-Gal4FF fusion can not only enhance the transcriptional activation efficiency of Gal4FF in the nucleus, but also avoid the overaccumulation of Gal4FF during embryonic development, ensuring that its transgenic line grows healthily to adulthood (Fig. 1D), without encountering developmental abnormalities caused by potential cytotoxicity (Fig. S3B,C). Based on this continuous fluorescent labelling strategy, we further generate the transgenic lines, enabling long-term visualised labelling specific to endodermal tissues and organs, with labelling capability extending into adulthood (Fig. 4A). The susceptibility of UAS to methylation is a pervasive issue (Goll et al., 2009; Pang et al., 2015). As zebrafish age, the transgenic lines exhibited a partial loss of cell labelling (Fig. 4B). In future, introducing insulator elements upstream and downstream of UAS cassettes or designing methylation-resistant UAS sequences could serve as potential solutions worth exploring.

In terms of application, the perpetual cycling Gal4-UAS system can provide an additional option and supplement to the few currently available visualised lineage-tracing methods, and it can also be combined with the Cre-loxP system for intersectional control of reporter gene expression to enhance specificity and flexibility in cell labelling (Satou et al., 2013; Tabor et al., 2019). In addition to fundamental biological research, the zebrafish transgenic lines with endoderm-specific and continuous fluorescent labelling can function as a visualisation tool for preclinical drug development, such as antiviral or antibacterial drug screening in the respiratory and digestive systems (Rampes and Ma, 2023; Habjan et al., 2024).

## MATERIALS AND METHODS

### Ethics statement

All zebrafish were maintained and raised under standard laboratory conditions according to Institutional Animal Care and Use Committees (IACUCs) protocols. This work is approved by the Animal Ethics Committee of Southwest University, Chongqing, China (ETHICS CODE Permit NO. IACUC-20240612-01).

### Zebrafish strains

The *Tg(hsp70l:Gal4FF)^cq185^*, *Tg(sox17:Gal4FF-T2A-EGFP)^cq186^* and *Tg(5×UAS:NLS-Gal4FF-PEST-T2A-EGFP)^cq187^*, abbreviated as *Tg(5×UAS:NP-Gal4FF-T2A-EGFP)*; *Tg(hsp70l:NLS-Gal4FF-PEST)^cq210^*, abbreviated as *Tg(hsp70l:NP-Gal4FF)*; *Tg(hsp70l:Cre-P2A-Gal4FF)^cq211^*; *Tg(sox17:Gal4FF)^cq212^*; *Tg(sox17:CreER^T2^)^cq213^*; *Tg(actb2:loxP-STOP-loxP-EGFP)^cq214^*; *Tg(5×UAS:EGFP)^nkuasgfp1a^* (Asakawa et al., 2008) and *Tg(sox17:GFP)^s870^* (Chung and Stainier, 2008 and Chung et al., 2008) transgenic lines were established and/or used in this study. Embryos were treated with 0.003% 1-phenyl-2-thiourea (PTU, Sigma) from 24 hpf to prevent pigmentation, as previously described (Yu et al., 2023). An equal number of male and female adult fish were used for experiments.

### Plasmid construction

The *NLS-Gal4FF-PEST-T2A-EGFP* fragment was constructed using Golden Gate cloning, as previously described (Jiang et al., 2023). The *Gal4FF* sequence containing an NLS domain, modified *PEST* with S440A mutation (Li et al., 1998) and *T2A-EGFP* sequence were spliced together through a one-pot restriction-ligation procedure. The *Cre-P2A-Gal4FF* fragment was constructed by joining the *Cre-P2A* sequence to the *Gal4FF* sequence using overlap PCR. Within the *pTol2* vector backbone: the *Gal4FF*, *NLS-Gal4FF-PEST* or *Cre-P2A-Gal4FF* fragment was subcloned the downstream of *hsp70l* promoter; the *Gal4FF*, *CreER^T2^* or *Gal4FF-T2A-EGFP* fragment was subcloned the downstream of *sox17* (-5.1k) promoter (Sakaguchi et al., 2006); the *NLS-Gal4FF-T2A-EGFP* or *NLS-Gal4FF-PEST-T2A-EGFP* fragment was subcloned the downstream of *CMV* promoter; and the *5×UAS* or *10×UAS* element, and the *loxP-STOP-loxP-EGFP* fragment was subcloned the downstream of *actb2* promoter. Within the *pcDNA3.1* vector backbone: the *Gal4FF* sequence was joined with the *ER^T2^* sequence using overlap PCR and then subcloned the downstream of *CMV* promoter; the *EGFP*, *EGFP-PEST* or *NLS-Gal4FF-PEST-T2A-EGFP* fragment was subcloned the downstream of *5×UAS* element. All primers and the component sequences used in constructs are detailed in Table S1.

### Generation of transgenic lines

The *pTol2-hsp70l:Gal4FF*, *pTol2-hsp70l:NLS-Gal4FF-PEST*, *pTol2-hsp70l:Cre-P2A-Gal4FF*, *pTol2-sox17:Gal4FF*, *pTol2-sox17:Gal4FF-T2A-EGFP*, *pTol2-sox17:CreER^T2^*, *pTol2-5×UAS:NLS-Gal4FF-PEST-T2A-EGFP* and *pTol2-actb2:loxP-STOP-loxP-EGFP* constructs were used for transgenesis. Each construct was co-injected with 50 pg of capped Tol2 transposase mRNA into zebrafish embryos of the AB genetic background at the one-cell stage. The *Tg(5×UAS:NLS-Gal4FF-PEST-T2A-EGFP)* founders with germline transmission were identified through outcrossing with the *Tg(sox17:Gal4FF-T2A-EGFP)* lines. The identified founders were then crossed back to the AB genetic background for three consecutive generations to screen the optimal and stable lines.

### Cell culture, transfection and induction

Human embryonic kidney (HEK) 293T cells were purchased from Cell Bank, Chinese Academy of Sciences (Shanghai, China). The cells were pre-plated in six-well plates and cultured in DMEM medium (Gibco) containing 10% fetal bovine serum (Gibco) and 1% penicillin-streptomycin (Gibco). Upon reaching 70-80% confluency, *pcDNA3.1-CMV:Gal4FF-ER^T2^* plasmids were co-transfected with *pcDNA3.1-5×UAS:EGFP*, *pcDNA3.1-5×UAS:EGFP-PEST* or *pcDNA3.1-5×UAS:NLS-Gal4FF-PEST-T2A-EGFP* plasmids into the cells. Transfection was carried out using Lipofectamine 3000 (Invitrogen, L3000-001) according to the manufacturer's instructions. For conditional induction of Gal4FF-ER^T2^ activity, post-transfection cells were treated with 1 μM tamoxifen (Sigma) for 24 h and then cultured until the time-points of analyses.

### Heat-shock treatment

Different fluorescent reporter lines with *Tg(hsp70l:Gal4FF)* or *Tg(hsp70l:NLS-Gal4FF-PEST)* genetic background at 12 hpf were heat-shocked at 38.5°C for 30 min, and then incubated at 28.5°C until analyses at the larval stage or raised until adulthood. The fluorescent reporter lines with the *Tg(hsp70l:Cre-P2A-Gal4FF)* genetic background at 24 hpf were heat-shocked at 38.5°C for 10, 20, 30 or 40 min separately, and then incubated at 28.5°C until the collective analysis 180 min later.

### Tamoxifen treatment

For conditional induction of CreER^T2^ activity, the double transgenic embryos *Tg(sox17:CreER^T2^; actb2:loxP-STOP-loxP-EGFP)* were treated with 5 μM tamoxifen (Sigma). The treatment was initiated at 2 hpf, with embryos incubated at 28.5°C under either continuous exposure to tamoxifen until analysis at 12 hpf or exposure for 24 h and further incubation until analysis at 96 hpf.

### Southern blotting

Genomic DNA was isolated from *Tg(5×UAS:NLS-Gal4FF-PEST-T2A-EGFP)* embryos. 10 μg of genomic DNA were digested with 150 units of the EcoRI (NEB) restriction enzyme. Southern blotting was performed as previously described (Green and Sambrook, 2021). The digoxigenin-labelled RNA probe, synthesised from a 402 bp DNA fragment of Gal4FF, was used for hybridisation. Primers used for probe synthesis were as follows: forward, 5′-CTGTCTTCTATCGAACAAGCATGCG-3′; reverse, 5′-ATTTAGGTGACACTATAGACTACTCTCTTCCGATGATGATG-3′ (the SP6 promoter sequence is underlined).

### Fluorescence imaging

Transgenic embryos were mounted in 35 mm glass bottom dishes containing 0.6-1% low melting point agarose as previously described (Yang et al., 2021). Images were captured using a 20× water immersion objective on the LSM880 confocal microscope (Zeiss). Three-dimensional images and stitched images were generated by *z*-stacks and Tile Scan, respectively, using ZEN2010 software (Zeiss).

Adult zebrafish imaging was performed as previously described (Gupta and Mullins, 2010). Anaesthesia was administered using 140 mg/l Tricaine (MS-222, sigma), followed by an abdominal dissection to remove the skin and body wall muscles, exposing the internal organs. Images were captured using a 1× plan apochromatic objective on the Leica M205 FCA stereo microscope.

### Fluorescence *in situ* hybridisation and antibody staining

Whole embryos or tissues were fixed overnight in 4% formaldehyde solution. Tissues were embedded in optimal cutting temperature (OCT) compound (Sakura) and frozen sectioned at 15 µm using a Leica CM 1950 cryostat. Fluorescence *in situ* hybridisation (FISH) was performed as previously described (He et al., 2020; Yang et al., 2022) using *bhmt*, *prss1* and *fabp2* antisense probes labelled with digoxigenin, anti-digoxigenin-POD (1:1000, Roche, 11633716001), and the tyramide signal amplification and fluorescence detection system (TSA, PerkinElmer).

Antibody staining was performed as previously described (Wu et al., 2023) using anti-GFP (1:1000, Invitrogen, A11122) primary antibody and Alexa 488-conjugated donkey anti-rabbit IgG (1:1000, Invitrogen, A-21206) secondary antibody. The nuclei were stained with the DNA fluorochrome 4′,6-diamidino-2-phenylindole (DAPI, Sigma).

### Quantification and statistical analysis

Cell Counting, Co-localisation Threshold plug-ins, Look-up table (LUT)-based colour mapping and Fluorescence Image Analysis tool in ImageJ (version 1.50d) were used to quantify the ratios of EGFP-labelled cells and the mean fluorescence intensity of specific tissue regions. All sampling was carried out using randomly assigned siblings. Data were analysed for statistical significance in GraphPad Prism (version 9.0.0) using comparison of means, one-way ANOVA with Tukey's multiple comparisons test, a Chi-square test or an unpaired two-tailed Student's *t*-test. Variance for all groups of data are presented as ±s.d. No data were excluded from analyses. The exact sample size (*n*), *P*-value summarised with asterisks (*) or hashes (^#^), and statistical tests are indicated in the figures and figure legends.

## Supplementary Material

10.1242/develop.204289_sup1 Supplementary information

Table S1. Component sequences and primers used in constructs. The component sequences list *5×UAS*, *10×UAS*, and *CMV* promoters, *NLS* and *PEST* domains, *Gal4FF*, *T2A*, *Cre*, *ER^T2^*, *loxP-STOP-loxP*, and *EGFP* elements, along with their corresponding nucleotide sequences. The primers are designed for cloning *hsp70l*, *sox17*, and *CMV* promoters, and for PCR amplification of *NLS-Gal4FF*, *PEST*, *T2A-EGFP*, *Cre-P2A*, *ER^T2^*, and *loxP-STOP-loxP* elements, with both forward and reverse sequences provided for each.
