# Supplementary material for: Long-term labelling and tracing of endodermal cells using a perpetual cycling Gal4-UAS system
Source: Development. 2025 Mar 21;152(6):dev204289. doi: 10.1242/dev.204289 (PMC11959616; doi:10.1242/dev.204289)
Supplement: Supplementary information [file develop-152-204289-s1.pdf]

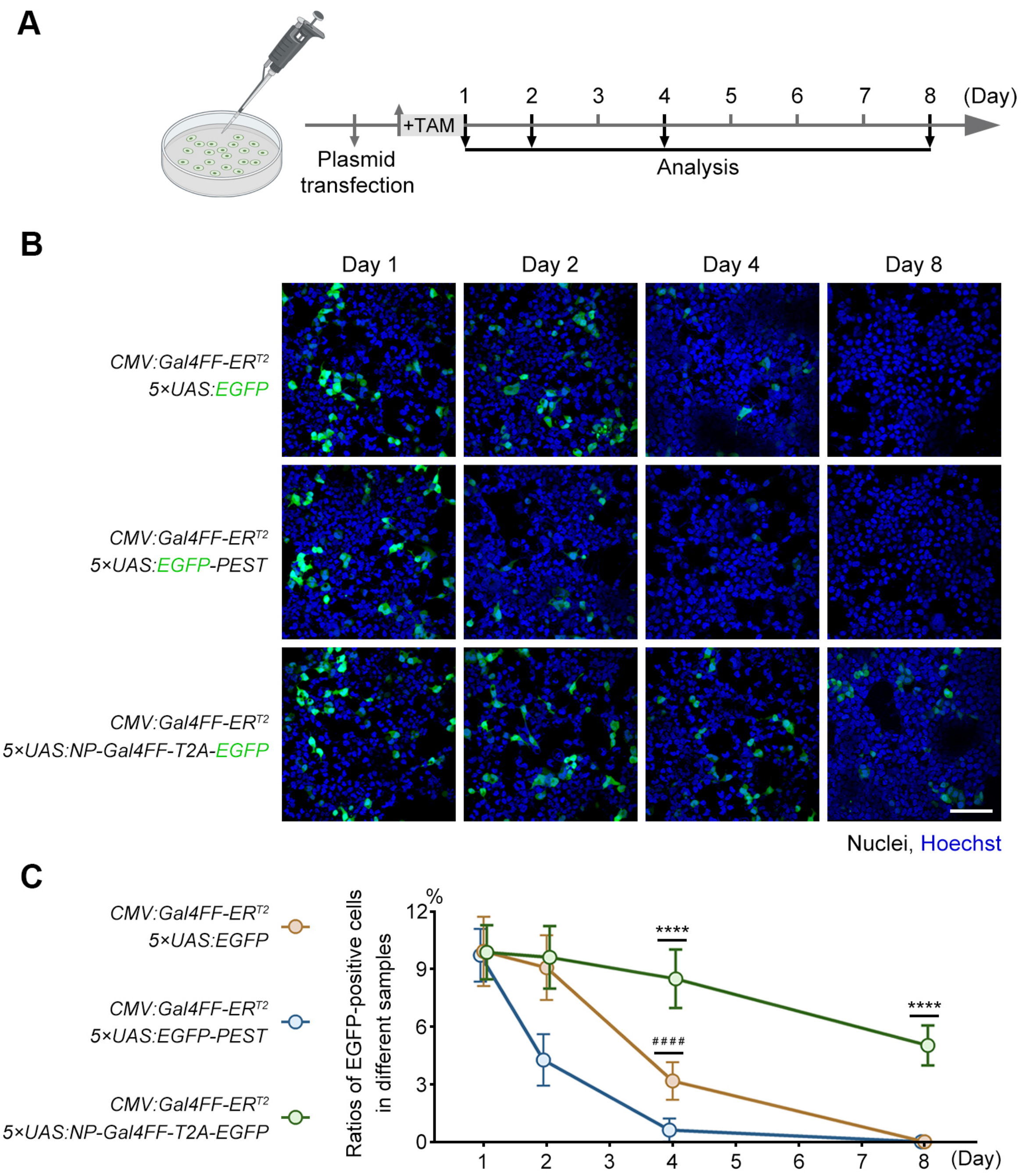

Fig. S1. Testing the perpetual cycling Gal4-UAS system in 293T cells.

(A) Experimental timeline for transfection, induction and analysis of 293T cells. The *CMV:Gal4FF-ER*^{T2} construct was transiently co-transfected with *5×UAS:EGFP*, *5×UAS:EGFP-PEST* or *5×UAS:NP-Gal4FF-T2A-EGFP* constructs into 293T cells,

Development • Supplementary information

followed by 24-hour treatment with tamoxifen (TAM), and analyses at Days 1, 2, 4, and 8. (B) Cells transfected with *5×UAS:EGFP-PEST* showed faster EGFP signal decay, while those transfected with *5×UAS:NP-Gal4FF-T2A-EGFP* showed prolonged EGFP signal duration. Scale bar, 50 μm. (C) Statistical graph of ratios of EGFP-labelled cells in these samples (per group *n*=10). Data are expressed as mean±SD, One-way ANOVA with Tukey's multiple comparisons test. ****P<0.0001, *CMV:Gal4FF-ER*^{T2}*/5×UAS:NP-Gal4FF-T2A-EGFP* vs *CMV:Gal4FF-ER*^{T2}*/5×UAS:EGFP*; ####P<0.0001, *CMV:Gal4FF-ER*^{T2}*/5×UAS:EGFP* vs *CMV:Gal4FF-ER*^{T2}*/5×UAS:EGFP-PEST*.

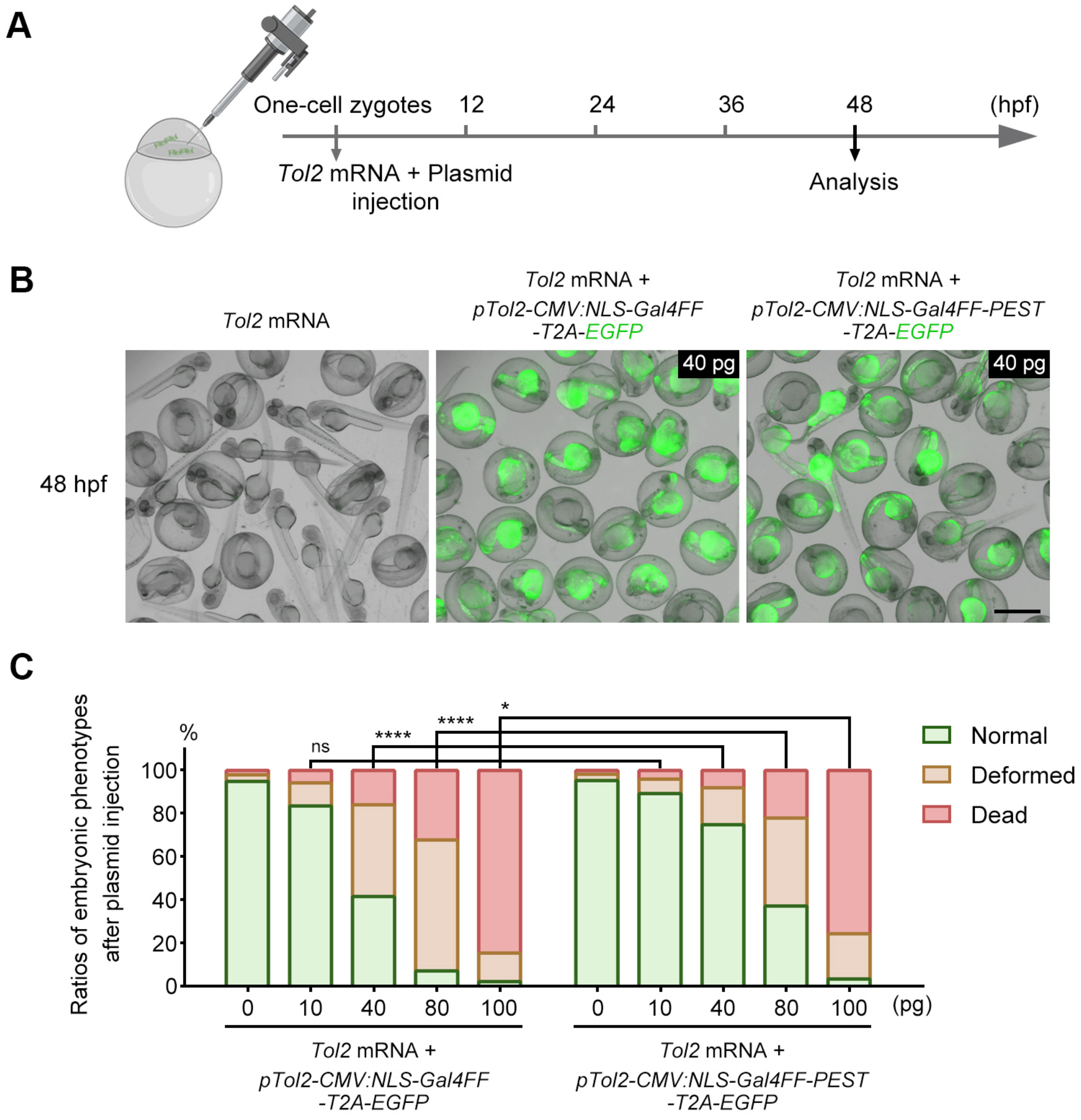

Fig. S2. Toxicity assay through plasmid injection into zebrafish embryos.

(A) Experimental timeline for plasmid injection and analysis of zebrafish embryos. The *Tol2* mRNA was co-injected with *pTol2-CMV:NLS-Gal4FF-T2A-EGFP* or *pTol2-CMV:NLS-Gal4FF-PEST-T2A-EGFP* constructs into one-cell zygotes and analysed at 48 hpf. (B) Phenotypic comparison of embryos at 48 hpf following injection of different plasmids at a dose of 40 pg. Scale bar, 2 mm. (C) Statistical graph of ratios of dead and deformed embryos after injection of different plasmids at doses of 0, 10, 40, 80, and 100 pg (per group n=230). Chi-square test. ns, not significant; ****P<0.0001; *P=0.0488.

Development • Supplementary information

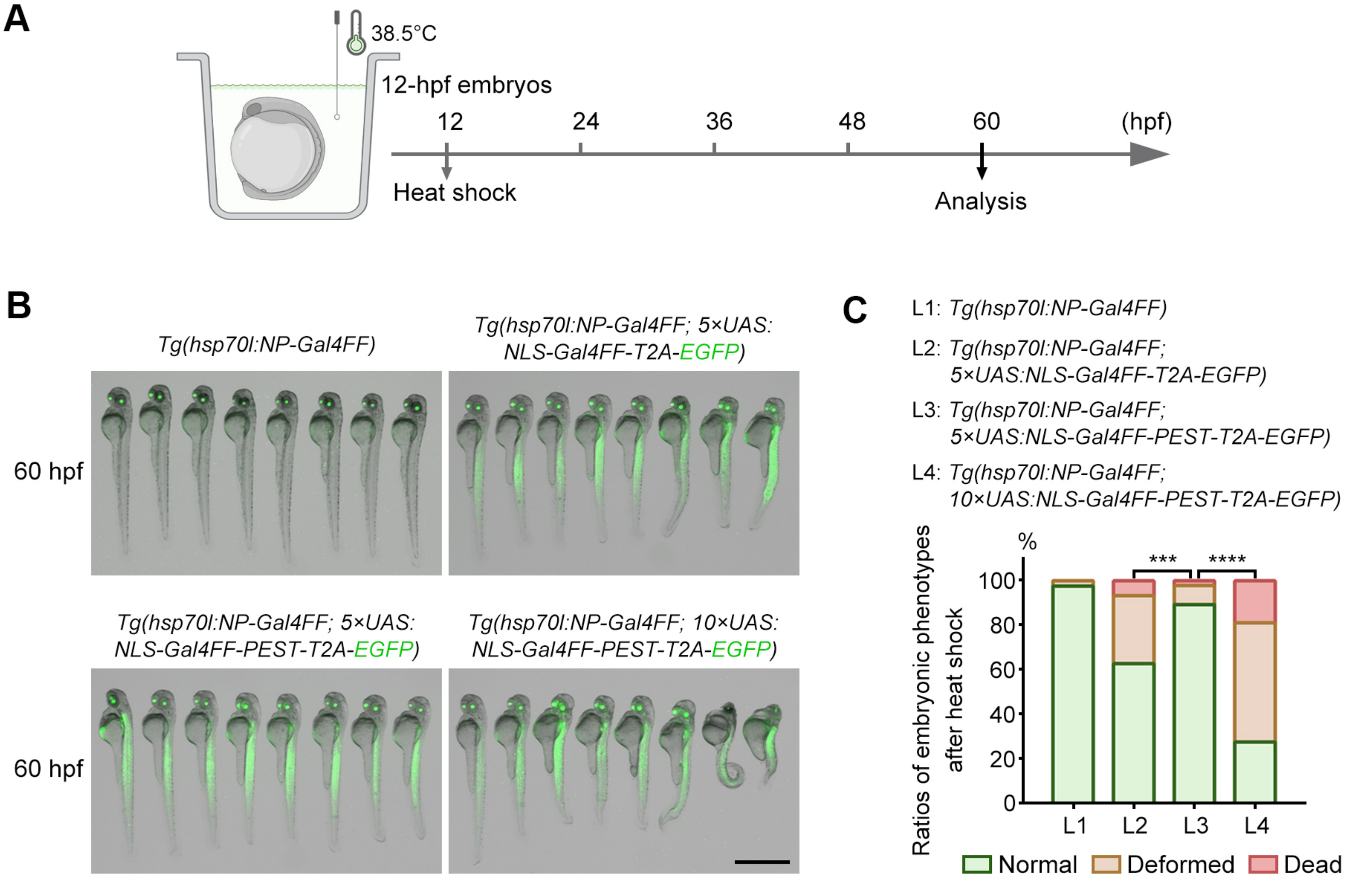

Fig. S3. Toxicity assay through heat-shock induction of zebrafish embryos.

(A) Experimental timeline for heat-shock induction and analysis of zebrafish embryos. The *Tg(hsp70l:NP-Gal4FF)*, *Tg(hsp70l:NP-Gal4FF; 5×UAS:NLS-Gal4FF-T2A-EGFP)*, *Tg(hsp70l:NP-Gal4FF; 5×UAS:NLS-Gal4FF-PEST-T2A-EGFP)*, or *Tg(hsp70l:NP-Gal4FF; 10×UAS:NLS-Gal4FF-PEST-T2A-EGFP)* transgenic lines were heat-shocked at 12 hpf and analysed at 60 hpf. (B) Phenotypic comparison of different transgenic embryos at 60 hpf following heat-shock induction. Scale bar, 2 mm. (C) Statistical graph of ratios of dead and deformed embryos under different transgenic backgrounds after heat-shock induction (per group n=85). Chi-square test. ****P<0.0001; ***P=0.0002. L1, L2, L3, and L4 represent different transgenic lines as indicated.

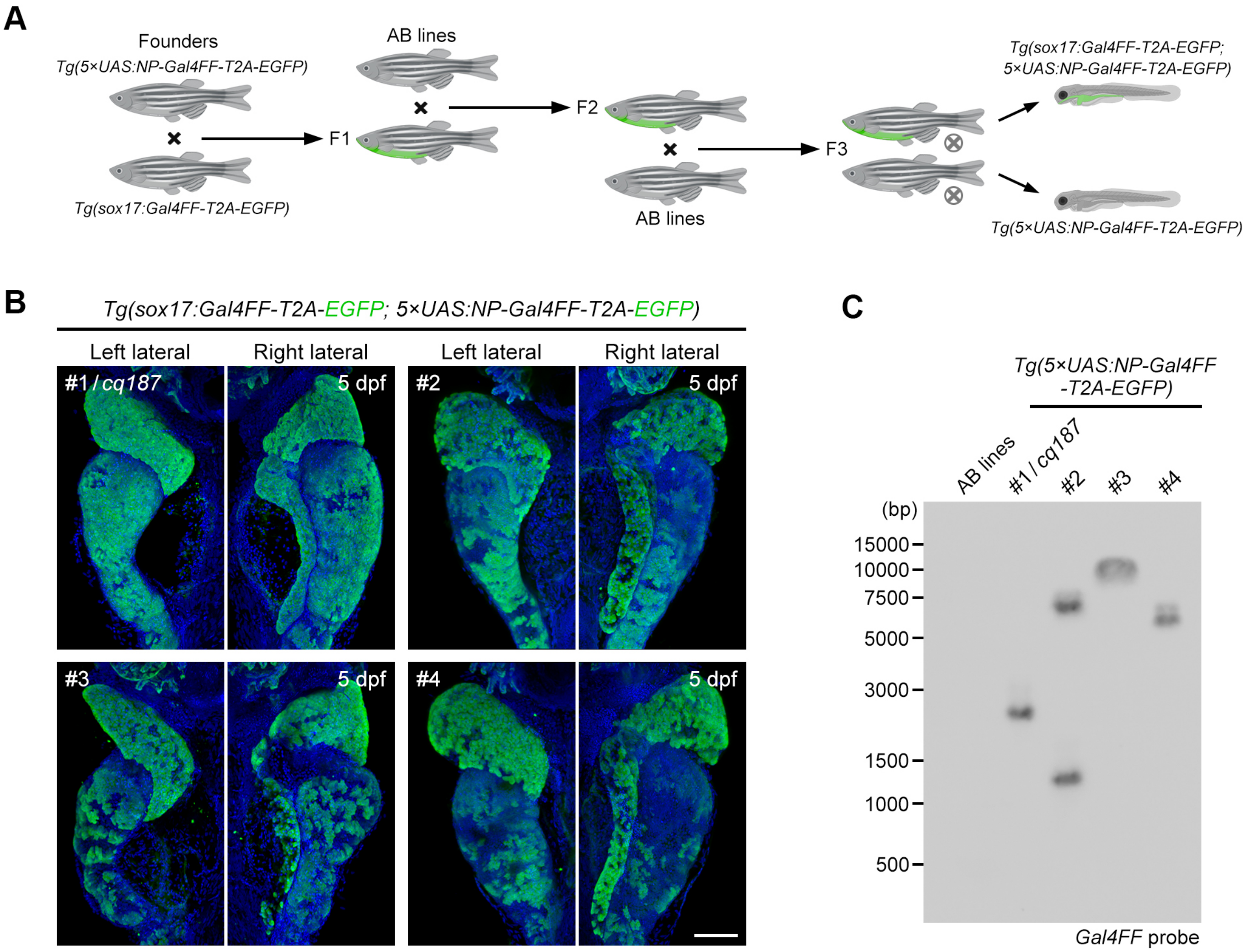

Fig. S4. Screening and identification of endoderm-specific labelling lines.

(A) Schematic of screening stable lines of transgenic zebrafish. The *Tg(5×UAS:NP-Gal4FF-T2A-EGFP)* founders were identified by outcrossing with the *Tg(sox17:Gal4FF-T2A-EGFP)* lines, and their offspring were crossed back to the AB genetic background for three consecutive generations to screen the optimal and stable lines. (B) Comparison of EGFP expression patterns at 5 dpf in offspring of different founders (#1, #2, #3, #4). Scale bar, 100 μm. (C) Southern blot analysis of genomic DNA isolated from offspring of different founders. Genomic DNAs were digested with *Eco*RI and hybridised with the *Gal4FF* probe.

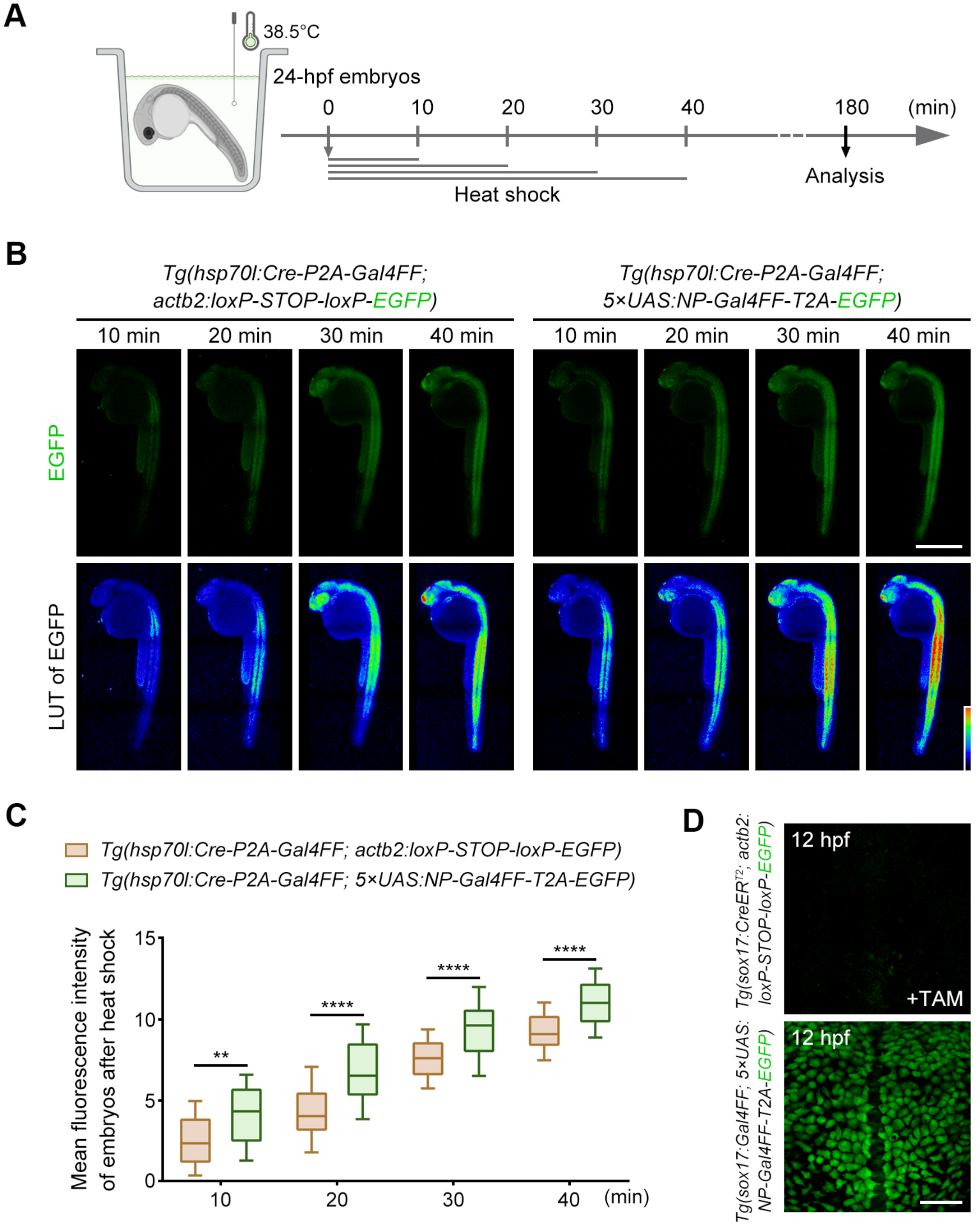

Fig. S5. Comparison of perpetual cycling Gal4-UAS system and Cre-loxP system.

(A) Experimental timeline for heat-shock induction and analysis of zebrafish embryos. The *Tg(hsp70l:Cre-P2A-Gal4FF; actb2:loxP-STOP-loxP-EGFP)* and *Tg(hsp70l:Cre-*

Development • Supplementary information

P2A-Gal4FF; 5×UAS:NP-Gal4FF-T2A-EGFP) transgenic lines were heat-shocked at 24 hpf for 10, 20, 30 or 40 minutes (min) separately, and collectively analysed 180 minutes later. (B) Fluorescent images of transgenic embryos under different heat-shock durations (B, scale bar, 500 μm). A look-up table (LUT) was used to map the EGFP fluorescence intensity. (C) Statistical graph of mean fluorescence intensity of the indicated transgenic embryos under different heat-shock durations (per group n=20). Data are expressed as mean±SD, Student's t-test. ****P<0.0001; **P=0.0023. (D) Comparison of EGFP expressions in *Tg(sox17:CreER^{T2}; actb2:loxP-STOP-loxP-EGFP)* and *Tg(sox17:Gal4FF; 5×UAS:NP-Gal4FF-T2A-EGFP)* transgenic embryos at 12 hpf (D, scale bar, 100 μm). Tamoxifen (TAM) treatment of embryos was initiated at 2 hpf and continued until 12 hpf for analysis.

Development • Supplementary information

Table S1. Component sequences and primers used in constructs.

The component sequences list *5×UAS*, *10×UAS*, and *CMV* promoters, *NLS* and *PEST* domains, *Gal4FF*, *T2A*, *Cre*, *ER^{T2}*, *loxP-STOP-loxP*, and *EGFP* elements, along with their corresponding nucleotide sequences. The primers are designed for cloning *hsp70l*, *sox17*, and *CMV* promoters, and for PCR amplification of *NLS-Gal4FF*, *PEST*, *T2A-EGFP*, *Cre-P2A*, *ER^{T2}*, and *loxP-STOP-loxP* elements, with both forward and reverse sequences provided for each.

Available for download at
https://journals.biologists.com/dev/article-lookup/doi/10.1242/dev.204289#supplementary-data
